# Feeding rate in adult *Manduca sexta* is unaffected by proboscis submersion depth

**DOI:** 10.1371/journal.pone.0302536

**Published:** 2024-05-29

**Authors:** Tomas F. Pierce, Tyson L. Hedrick

**Affiliations:** Dept. of Biology, University of North Carolina at Chapel Hill, Chapel Hill, NC, United States of America; Northwestern University, UNITED STATES

## Abstract

Adult moths from framily *Spingidae* (i.e. hawkmoths or sphinx moths) commonly feed on flower nectar through an extended proboscis, often several centimeters in length and longer than the body of the moth. Feeding on a viscous liquid (nectar) through a long and narrow tube is a challenging fluid dynamic problem and the subject of long-running scientific investigation. Here we characterized the relationship between proboscis submergence depth and nectar drinking rate in *Manduca sexta* hawkmoths. Video recordings of moth feeding bouts were collected and neural networks were used to extract data by object localization, tracking the location of the nectar meniscus and moths’ proboscis tips. We found that although feeding rates vary among bouts, the variation was not associated with proboscis submergence depth. These results show that despite the theoretical possibility of fluid uptake through the walls of the proboscis, such effects do not have a substantial effect on nectar uptake rate, and suggest that nectar must traverse the full length of the proboscis.

## Introduction

Many adult moths, including *Manduca sexta* [1] feed on flower nectar ingested using a long tube-like organ called a proboscis. However, members of Sphingidae including *M. sexta* are atypical among moths for feeding in hovering flight. The high metabolic power requirements of hovering flight [2] may prioritize rapid nectar uptake, and previous work on *Macroglossum stellatarum* hawkmoths indicates a nonlinear relationship between feeding rate, sucrose concentration of nectar, and nectar viscosity [3]. The volume of nectar ingested by *Manduca sexta* hawkmoths and their preferred sucrose concentration is also influenced by relative humidity [4, 5]. Geometric and structural features of the feeding assembly of proboscis and internal sucking pump also influence feeding rate in *M. sexta* and other species [6]. Other research has been focused on the factors that induce feeding and influence flower selection [7], which include visual [8] and olfactory [9] cues. Depending on relative humidity and time elapsed since eclosure, average daily nectar ingestion for *Manduca sexta* hawkmoths varies between 0 μL and 500 μL of nectar fluid [4]. To our knowledge, no extant published work addresses whether proboscis submergence depth influences nectar consumption rate. However, the depth to which *M. sexta* moths submerge their proboscis varies within and between recordings of feeding events, and recently published theoretical analysis of Lepidopteran mouthpart achitecture suggests that fluid uptake may occur along the length of the proboscis [10]. This suggests a possible increase in the feeding rate through a deeply submerged proboscis based on a model where transport of nectar through the proboscis has an energy cost proportional to travel distance, and that greater submergence would permit a shorter travel distance that decreased energy costs, increasing nectar ingestion rate. Thus, we hypothesized that the feeding rate would be positively influenced by the proboscis submergence depth of the moths.

Prior art on the measurement of proboscis position assumes a top-down view and restrained subjects [11], making it unsuitable for characterizing the feeding behavior of freely moving insects. As part of this project, software tools based on machine learning methods were developed to extract positional information from video data.

Machine learning (ML) technologies are of increasing utility for scientific research [12]. ML refers to the use of automated data analysis methods which can detect patterns in data with minimal or without human intervention and make predictions from detected patterns [12]. Convolutional neural networks (CNNs) are a ML technology suitable for image analysis type tasks that utilizes sequential learned convolutional functions to approximate a function [13]. The project utilizes a CNN to locate meniscus positions and proboscis positions in video frames. The architecture used for localization is an image-to-image U-net [14] implemented using Keras [15, 16]. The U-net architecture has the useful property of being trainable from a relatively small collection of examples [14], which limits the amount of labeling work required to produce an effective object location extractor. By locating the meniscus of a column of nectar fluid in each frame, the project is able to compute the approximate rate of nectar consumption from the derivative of a fitted LOESS [17] model for 43 of 55 video files. Determining the factors influencing the rate of nectar consumption in M. sexta hawkmoths may have applications in other fields, and may illuminate biomechanical constraints underpinning Lepidopteran nectar ingestion.

Despite our success in applying machine learning technologies to measure the feeding rate of unrestrained *Manduca sexta* hawkmoths, our results do not support our hypothesis and feeding rate was uncorrelated with proboscis submersion depth.

## Materials and methods

The overall approach had two phases, the experimental phase and the computational phase. During the experimental phase, we collected videos of M. sexta hawkmoths feeding from an artificial flower. During the computational phase, we extracted positional measurements of relevant features from the video recordings. Data were visualized using the matplotlib [18] and seaborn [19] libraries.

### Experimental

Eleven male *Manduca sexta* moths were obtained as pupae from the colony at the University of North Carolina at Chapel Hill (UNC) and allowed to eclose in 20x20x30 cm mesh cages maintained with a 20:4 light:dark cycle. Only male moths were used to control for sex-specific behavior such as oviposition. Moths in mesh cages were provided with water *ad libitum*, and began feeding experiments three to four days post-eclosure. Moths were uniquely identified and several feeding recordings were typically recorded over a several day span.

**Fig 1:**
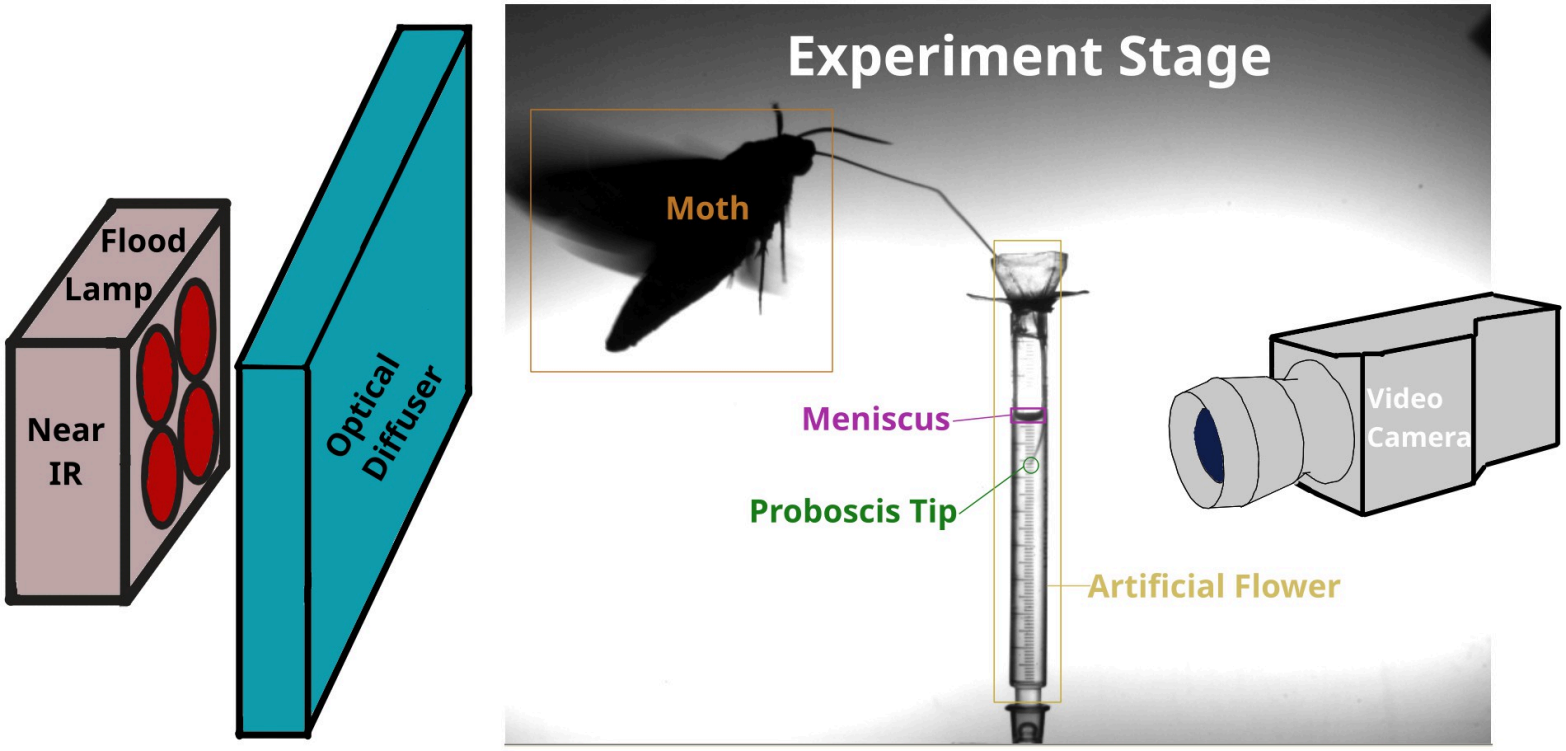
Experiment setup. Moths were filmed using a Vision Research Phantom camera and lit from behind using near-IR LED lamps and an optical diffuser to maximize contrast. This example image has been labeled manually. Moths fed either while flying or while standing on a perch (not shown).

To record a moth feeding bout, the moth was first weighed using an electronic balance then placed in the experimental flight cage, which had clear glass on top and three sides, with a black plastic surface beneath. The remaining side was covered with a mosquito netting curtain to permit experimenter access. As shown in the Fig 1 schematic, videos were recorded using a Phantom v7.1 camera (Vision Research, Wayne, NJ) configured to continuously record a lateral view of an artificial flower constructed from 1 ml syringe body at 10 frames per second (FPS). Opposite the camera and behind the nectar source, an optical diffuser plate and multiple near-IR (680 nm wavelength) LED lamps provided illumination from behind the experimental scene. This near-IR wavelength is detectable by the camera but not expected to be visible to the moths, which use blue, green, and UV photo-receptors [20] that the moths use for sight [21] and color vision. However, the possibility that the moths detect some of the near-IR light was not directly tested here.

The artificial flower was filled with a solution of 1:4 honey:water artificial nectar, and the IR LEDs carefully positioned to minimize shadows in the artificial flower tube. A funnel cap was attached to the top of the syringe body to guide in the moth’s proboscis into the tube.

If the moth did not begin flight warm-up behavior shortly after introduction to the recording cage, such behavior was encouraged by exposing the moth to bergamot oil scent and gently squeezing the moth’s abdomen. Once the moth was flying, the artificial flower tube was moved into the moth’s field of view until the moth extended its proboscis and initiated feeding behavior. Once the moth initiated feeding from the artificial flower tube, the artificial flower tube was returned to its position on the recording stage slowly enough that the moth followed the motion of the artificial flower tube. After feeding behavior stopped, the camera recording was saved. If the moth continued to fly and exhibit feeding interest after the videos were saved, another recording was attempted or the moth was permitted to feed until satiated to maintain health. Once feeding ceased, the moth was removed, weighed, and returned to its holding chamber. In cases where the moths showed feeding interest but did not warm up to fly, they were positioned on a ring stand near the flower and allowed to feed while perched. A total of 55 recordings were collected from the eleven moths. Each moth produced between 1 and 15 recordings, averaging 4.5 recordings per moth. Of the 42 recordings analyzed, the recording duration varied between 20 seconds and 589 seconds, with an average duration of 185 seconds. Details about recording duration and other metadata can be found in S1 Table.

### Computational

To perform the analysis, it was necessary to extract numeric data from video files. To that end, a custom software pipeline was developed to automatically crop the video to the region of interest, employ neural networks for object localization of the nectar meniscus and proboscis tip, and calculate nectar ingestion rate and proboscis submergence from these results.

#### Restriction and cropping

The first stage in the computational approach was to restrict attention to the area of interest. To do this, the software found the column of pixels with the highest density of horizontal edges and cropped to the 100 pixel columns within 50 pixels of that column. This cropping strategy consistently produced a 600 x 100 pixel sub-region containing the artificial flower due to the volume markings on the syringe from the original 600 x 800 pixel frame. The density of horizontal edges in a column were found by using scikit-image [22] to apply a horizontal Sobel transform [23], before a threshold by Li’s method [24] was applied to the absolute magnitude of the horizontal edges to produce a mask, which was then summed by column to count the number of horizontal edges in each column.

#### Localization

**Fig 2:**
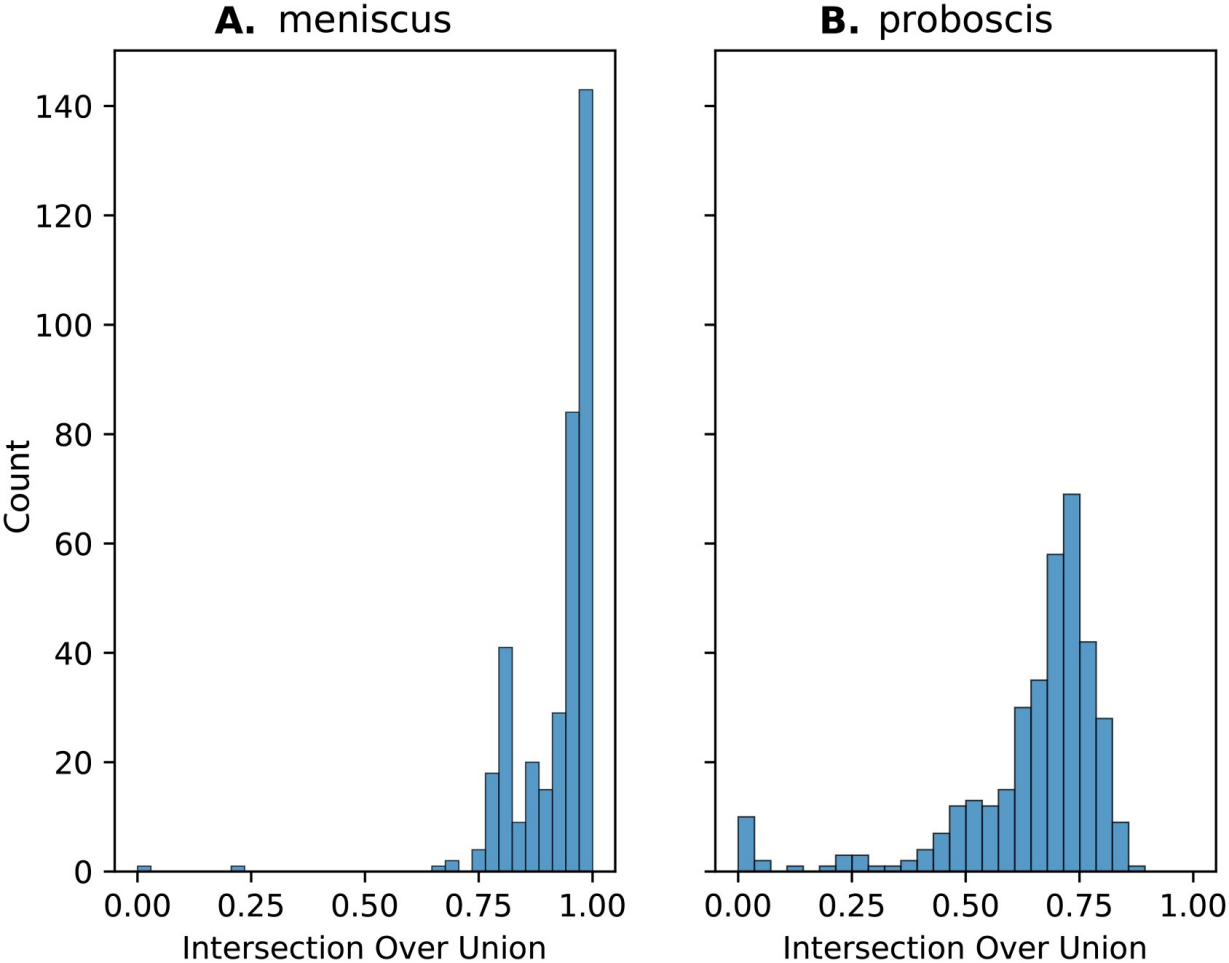
Test performance of object-locating neural networks. A: Meniscus network performance. B: Proboscis network performance. Histogram of intersection-over-union measurements for meniscus-tracking and proboscis-tracking neural networks on a test set randomly selected from the frames in the data set.

The cropped images were used to locate the proboscis of the moth and the meniscus of the nectar fluid column. Two convolutional neural networks of the same architecture —shown in S1 Fig —were then used to extract the meniscus position and the proboscis position. The architecture in use is a U-net [14] for both networks. A training dataset of 850 images was randomly selected from the video library and hand labeled using the online CVAT.ai annotation tool [25]. The training dataset was subject to data augmentation to produce a much larger dataset. A test dataset of approximately 370 images was randomly selected from the video library and hand labeled using the online CVAT.ai annotation tool [25]. A weight decay parameter of 0.0005 was used to enhance training [26]. Performance was evaluated on the held out test set of approximately 370 images by calculating the intersection over union —a common performance metric [27] —of the neural network’s predictions against the test set (Fig 2).

When provided a batch of images, the networks produce an equally sized batch of prediction images where the output can be interpreted as the probability that the pixel is part of the object being located.

A threshold of 0.5 is used to convert the output into masks, and the scikit-image library is used to find the objects in the mask and compute their properties, using the measure.label and measure.regionprops functions included in scikit-image [22].

At this point, the approaches for meniscus and proboscis localization diverge. For meniscus location, the largest object in each mask was assumed to be the meniscus, and the object’s centroid was then added to the measurements table along with some geometric information. As this system will sometimes erroneously locate the meniscus, highly unusual measurements are presumed to be incorrect and filtered using an isolation forest originating from scikit-learn [28].

For proboscis location, the scikit-image library was used to find the bounding boxes of recognized objects. Because the proboscis can be partially obscured by shadows at the margin of the artificial flower tub, multiple objects might be recognized. The software selected the object with the bottom edge of its bounding box furthest from the top of the image for each time point in each recording.

**Fig 3:**
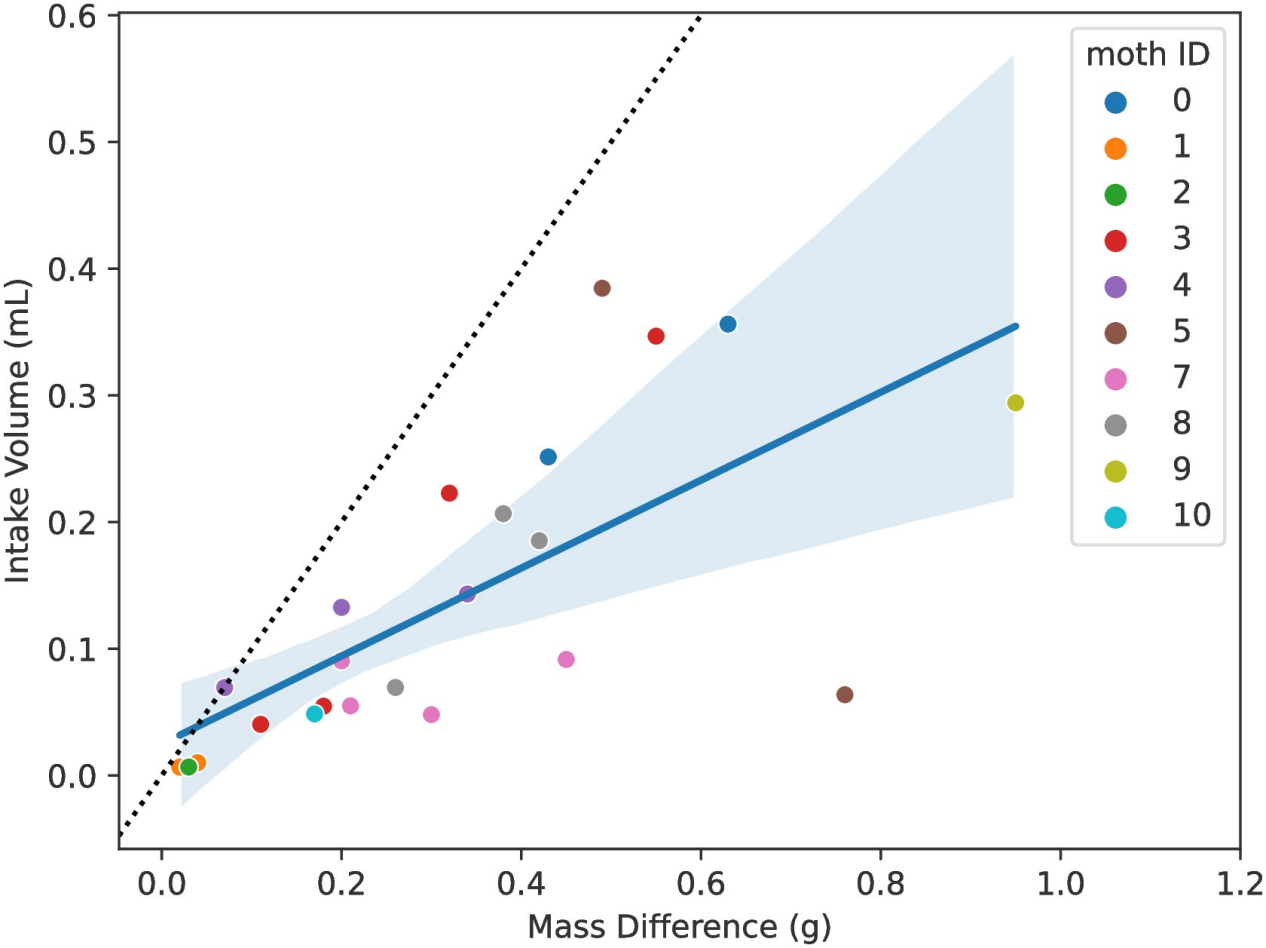
Measured nectar volume intake measurements approximate daily ingested nectar mass. Scatter plot of daily intake volume measured from neural network analysis of video data against daily mass change. Each point shows the total daily nectar volume and mass change for an individual moth. Mass intake and volume intake follow a significant (R = 0.7, p = 0.0002) linear relationship, with a slope less than 1. This is expected, because the mass measurement encompasses the entirety of a feeding bout whereas the video measurement captures an unknown portion of it. Regression line is in solid blue with a shaded 95% confidence interval, a 1-to-1 line is plotted in black dashes.

To check the correctness of the meniscus measurements, we compared the nectar intake implied by the network’s extracted measurements with mass measurements taken with an electronic balance. Results of this comparison (Fig 3), reveal that the mass intake is linearly correlated with the volume intake measured. Furthermore, the volume measurement never leads to overestimating the mass intake. Instead, volume measurements underestimate mass changes, the expected result because of additional moth feeding time that occurred before or after the recorded bout.

#### Drinking rate

Once meniscus positions were extracted, the meniscus rate of movement was calculated. Attempting to calculate it directly from the raw data produced variance in excess of the signal. Instead, a LOESS model [17] was computed from the meniscus positions and a derivative is approximated from the LOESS model. The loess_1d.loess_1d function from the loess package version 2.1.2 on PyPi [17] was used to perform curve fitting with default parameters of frac = 0.5 degree = 1, meaning that at each point, a 1-degree polynomial was fit using the closest $\frac{1}{2}$ of the available measurements in the recording with importance weighted using a Gaussian function of the distance from that point [17]. Testing with alternate parameter sets of (frac = 0.9, degree = 1), (frac = 0.2, degree = 1), and (frac = 0.5, degree = 2) did not alter the statistical signficance or non-significance of the results reported here. By measuring the rate of movement of the meniscus, the project is able to approximate the drinking rate of the moths. Loss of nectar volume to evaporation is negligible, as another study has measured a maximum nectar evaporation of 200 μL per day at 20% relative humidity [4] and the longest recording duration in this study is less than 600 seconds, see S1 Table. The maximum nectar loss in any individual recording to evaporation is approximately 1.4*10^−3^ mL, which is negligible. The only non-negligible process that removes nectar is the moths’ ingestion of the fluid.

Rate measurements are converted into units of milliliters per second by multiplication with a recording-specific conversion factor based on the syringe volume and pixel positions of volume markers.

#### Proboscis submergence

Proboscis submergence was calculated as the difference between the meniscus position and the proboscis tip, which is converted to millimeters. Under the assumption that *M. sexta* moths must submerge their proboscis to ingest nectar, data points in which the moth’s proboscis tip was above the meniscus and therefore outside the fluid column of the flower were not considered. Instead, the analysis is performed with the set of non-negative submergence measurements.

## Results

A library totaling 55 video files was collected totaling approximately 72 GB of video data. Both hover feedings and perched feedings were recorded. Custom software was used to automatically quantify nectar ingestion measurements and yielded results for 54 out of 55 recordings. However, 13 of the 55 recordings had properties that make successful analysis less likely, including being out of focus, having the experimenter visible, lack of drinking activity, lack of visible meniscus, or having an atypical artificial flower design.

The measured rate of nectar consumption was positive for 99.2% of rate measurements taken from the 42 videos that were more amenable to automated analysis, suggesting that fluid expulsion from the proboscis assembly is absent or incredibly rare, an unsurprising result.

**Fig 4:**
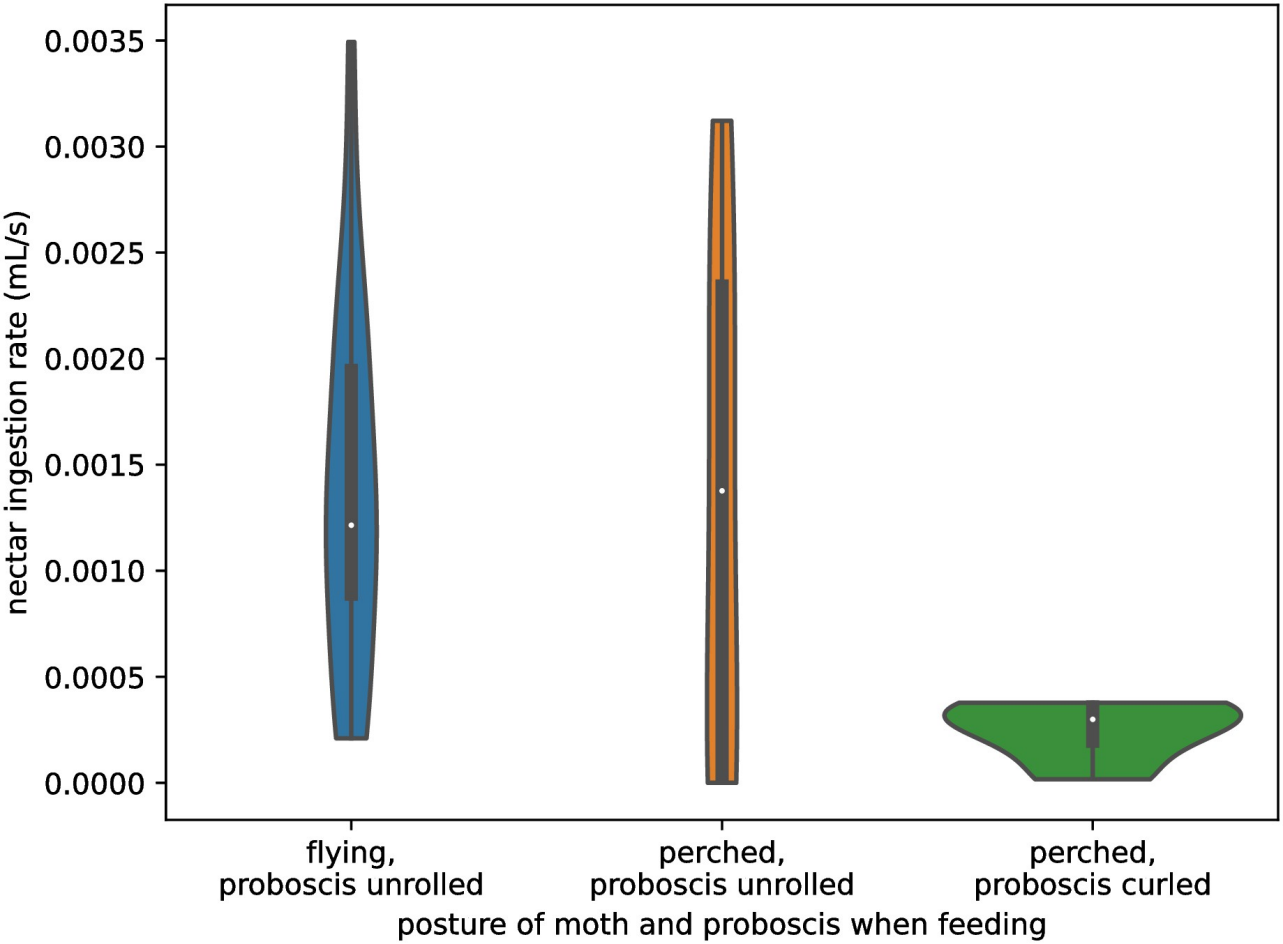
Effect of proboscis extension on feeding rate. A violin plot of drinking rate against feeding behavior, with each datapoint being one recording. Recordings with a curled proboscis tend to have significantly (p = 0.0007) lower drinking rates than flying feeding bouts with an extended proboscis by a Mann-Whitney U-rank test. When the moth is flying and feeding, only extended proboscis postures were observed.

As noted in Table 1, feeding behavior was not perfectly consistent among all trials. Five recordings showed a moth feeding from a fixed perch with a partially curled, not fully extended proboscis. This feeding mode is unlikely to be typical of wild individuals and while the moths did ingest some nectar through their curled proboscis, the rate was significantly less (p = 0.0007, Mann-Whitney U-rank test) than was observed in recordings of feedings where the moth fed in hovering flight with an extended proboscis (Fig 4). Recordings with a curled proboscis were therefore excluded from further analysis. Moths also occasionally fed through an extended proboscis while perched; feeding rate in this mode was not significantly different from recordings where the moth fed during hovering flight (p = 0.39, Mann-Whitney U-rank test; Fig 4).

**Table 1:** Recording and behavior differences.

	Hovering	Perched
Proboscis extended	29	8
Proboscis curled	0	5

Here we provide the breakdown of feeding behaviors and proboscis state during the 42 trials analyzed here.

During the course of the experiments, we also updated the flower design in an attempt to more readily and reliably elicit feeding behavior. To ensure that these changes do not confound our results we examined the data for temporal trends in feeding rate S2 Fig. This did not reveal any overall trend.

**Fig 5:**
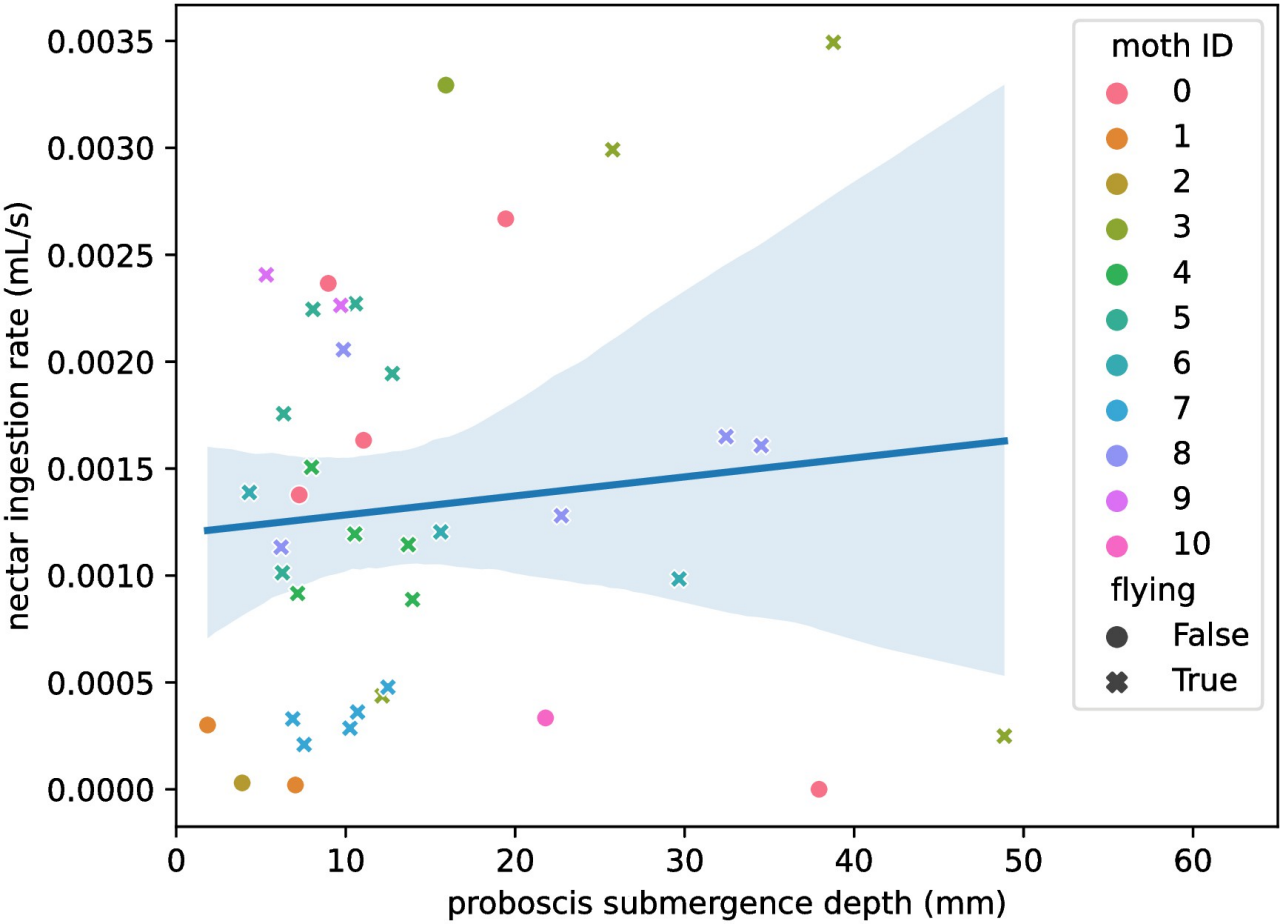
Nectar ingestion rate is independent of proboscis submergence. Scatterplot with superimposed linear model of per-recording median nectar ingestion rate against per-recording median proboscis submergence, with shaded 95% confidence interval. Each point shows the median submergence depth and median drinking rate for one recording. Points are colored by the moth’s ID and styled by whether the moth is perching (circle) or flying (x) in that recording.

Analysis of the remaining dataset of 38 video recordings from 11 moths revealed no significant correlation between feeding rate and proboscis submergence, as a linear regression of median submergence depth against median drinking rate yields (R = 0.27, p = 0.3; Fig 5). The left side of Fig 5 suggests the possibility of there being an effect in the region where proboscis submergence does not exceed 10 mm. This was investigated in S3 Fig by fitting a linear model to that subset of the data. This also produced a non-significant result (R = 0.42, p = 0.1) with no clear indication of non-linearity in residuals in S4 Fig. The independence of submergence depth and feeding rate suggests that the proboscis has little to no leakage or ability to absorb nectar at regions other than the tip.

Other notes on the experiments and analysis are as follows: LOESS models as used here are resistant to outliers, and produced negative rate estimates approximately 0.8% of the time. Negative feeding rates were deemed unlikely and ignored in analysis on the basis that they are expected to be artifacts of software failures. It may be suitable to adopt a monotonic local regression scheme instead on the expectation that fluid expulsion from the proboscis is not commonly expected or observed. LOESS curves also can fail to incorporate data if measurement density is low in a particular region, even if said regions lack major amounts of outliers. It may be more suitable to adopt a different K-nearest-neighbors regression to calculate the drinking rate on that basis.

The accuracy of the neural networks is limited by the quality of the data. Some frames occlude part of the proboscis organ, and obscuring the location of the proboscis tip makes getting an accurate location measurement difficult at best. If the meniscus of the fluid column in the flower is not visible in the video recording, then the rate cannot be computed. This helps explain why some of the recordings had to be excluded. Similarly, partial visibility due to insufficient lighting some recordings limit the achievable accuracy of proboscis tracking. If the moth’s proboscis is close to the edge of the tube, then the proboscis tip is sometimes obscured by shadows in that region. In an extreme case, in one of the discarded recordings the meniscus was never visible, and so produced no rate measurements. A full list of excluded recordings is provided in S2 Table.

Attempts to address these concerns, either via tests of alternate models for quantifying change in meniscus position over time or improvements to the neural networks did not yield any indication that further alterations to the analysis pipeline would alter the overall conclusions. Finally, although these considerations do suggest some limitations to automated or high-throughput quantification of feeding behavior in moths they could likely be further mitigated by using higher resolution or more sensitive cameras.

## Discussion

Adult moths of family *Sphingidae*, also known as sphinx moths or hawkmoths, feed in hovering flight by ingesting flower nectar through a long and narrow proboscis inserted into the flower and reaching the nectary. Taking up a viscous liquid such as nectar through a long and narrow tube is fluid dynamically challenging due to the high resistance of flow in this situation, leading to continued scientific investigation of how different insect groups accomplish this task, e.g. [29–31]. In hawkmoths, the hover-feeding mode further highlights these challenges because hovering is an energetically demanding flight mode [2], suggesting that the animals should maximize nectar uptake rate so as to minimize the duration of hovering [3]. As such, there has been much interest in how structural adaptations could speed nectar transport in the proboscis or otherwise reduce the needed feeding time, including a recent proposal that the lepidopteran (including hawkmoth) proboscis could fill through slits along the side, not just at the tip as in a drinking straw [10]. If this were the case, ingestion through upper submerged slits would help the moths reduce the amount friction that they need to overcome to ingest nectar due to the length of their proboscises [6], allowing more rapid nectar uptake. This proposal led us to hypothesize that proboscis submergence depth in *Manduca sexta* would be positively related to nectar feeding rate.

However, our results do not support our initial hypothesis. We found that the drinking rates of *M*. sexta hawkmoths were independent of the depth to which they submerged their proboscises. The independence is seen in all statistical tests and is especially clear when all data points are considered as in S5 Fig, which shows that a change in proboscis submergence was not associated with a change in nectar intake rate, and vice versa. Despite not supporting our initial hypothesis, our experimental result does not necessarily refute the importance of lateral slits as a fluid dynamic channel in the lepidopteran proboscis, because the independence of submergence depth and feeding rate would also arise if the rate-limiting step in feeding is unrelated to uptake at the proboscis. For example, if the rate at the muscular suction pump in the head is the limiting factor, proboscis submergence depth would not affect overall feeding rate even if deeper submergence promotes more rapid filling. However, in this case the lateral slits might still allow more effective short feeding bouts by reducing the time required for proboscis wetting. Additionally, our results do not contradict the possibility that only a specific region of the proboscis is involved in nectar uptake in *M. sexta* moths as has been observed for *Pieris rapae* butterflies [32], which would also prevent an association between proboscis submergence and nectar ingestion rate. Further experimental work to visualize fluid uptake around the proboscis while the moths feed from a fixed position, following [32].

Finally, our development of the camera and neural-network based feeding rate measurement system also provides an opportunity for further experimentation on lepidopteran feeding rate. Although we used it in an “offline” manner, making measurements from recorded video files, such an optical system could also function in an “online” mode, recording nectar levels continuously. This would permit direct measurement of how feeding rate varies during a feeding bout, potentially shedding light on the importance of different limiting factors in the nectar uptake process. One direction that could be studied are the effects of variables known to influence flower selection such as color and scents [33] or variables known to influence nectar preference and ingestion volume such as humidity and sugar concentration [4, 5] on rates of nectar ingestion and investigate whether the rate of nectar ingestion is also mediated by these factors or vice versa. It would also allow interactive experiments, where changes in nectar levels trigger mechanical, aerodynamic, or visual disturbances in the insect’s environment and thus probing how moths prioritize feeding over factors that might decrease the effective rate, increase the cost, or mimic a possible predatory threat. Additionally, the system could be used to investigate effects of altered properties of the nectar during a feeding bout on the effective rate of nectar uptake, such as a sudden increase in sugar concentration or anti-herbivorous-insect plant immune defense compounds.

## Supporting information

S1 Table The table summarizes the subset of collected videos that are used in the analysis. Measurements have been rounded for space concerns. Mass differences are per day, while all other measurements are per recording.
(PDF)

S2 Table Unsuitable recordings are excluded from analysis. The table summarizes the subset of collected videos that were excluded from the analysis and gives the reasoning for why they were excluded. Measurements have been rounded for space reasons. The entries that resemble duplicates originate from distinct recordings on the same day from different moths. Mass differences are per day, while other measurements are per recording.
(PDF)

S1 Fig Specifics of model architecture shown. Using the smaller cropped zone of data in the videos produces frames of dimensions 600 × 100, which required adjustments to the architecture relative to the examples given by prior [14] examples [15], which included a reduction in the number of skip connections, and changes to the number of convolution filters as an adjustment to available hardware.
(PDF)

S2 Fig Drinking rates are not influenced by date measured, and therefore independent of artificial flower design. Bottom row shows strip plots of all available data, top row shows line plots aggregating the same by moth ID. Left and right columns have been split to elide a period of several months where no data were collected. The date, and therefore the flower design, does not appear to have a consistent effect on the drinking rate of the moths. If the refinements had changed the behavior, we would expect to see general upward trends in the median drinking rate as the date progresses.
(PDF)

S3 Fig Submergence versus drinking rate remains independent when submergence depth is restricted to not exceed 10 mm. Points are colored by the moth ID for the recording and icons denote whether the moth flies in the recording or not. A linear model is drawn on the graph with a 95% confidence interval. Recordings with curled proboscises visible have been excluded. The model is dependent on the outlier at the bottom left, and excluding it causes the model to be statistically insignificant (p = 0.09 >0.05), for which the null hypothesis must be presumed.
(PDF)

S4 Fig No pattern in the residuals of drinking rate against submergence not exceeding 10 mm. Residuals of nectar ingestion rate are graphed against proboscis submergence depth.
(PDF)

S5 Fig No relationship between nectar ingestion rate and proboscis submergence depth exists in raw data. A scatterplot showing all measured proboscis submergences against all measured nectar ingestion rates by moth. As long as contact is made with the fluid, drinking can occur. The depth of submergence does not predict the rate of nectar ingestion, nor vice versa. Multiple nectar intake rates are observed at a given submergence depth, and multiple submergence depths are observed for almost any given intake rate.
(PDF)
