# Supplementary material for: Feeding rate in adult Manduca sexta is unaffected by proboscis submersion depth
Source: PLoS One. 2024 May 29;19(5):e0302536. doi: 10.1371/journal.pone.0302536 (PMC11135714; doi:10.1371/journal.pone.0302536)
Supplement: S1 Table — Measurements have been rounded for space concerns. Mass differences are per day, while all other measurements are per recording. (PDF) [file pone.0302536.s001.pdf]

moth ID	date	Recording Duration (s)	intake volume for recording (mL)	mass change for moth and date (g)	median probocsis submergence depth (mm)	median nectar ingestion rate (mL/s)
22	2022-01-26	200	0.36	N/A	11.23	0.0017
22	2022-01-31	70	0.15	N/A	19.45	0.0027
22	2022-02-01	130	0.17	0.43	7.18	0.0014
22	2022-02-01	42	0.09	0.43	8.95	0.0023
22	2022-02-08	589.3	0.16	N/A	2.04	0.0003
22	2022-02-09	589.3	0.13	N/A	0.64	0.0004
22	2022_02_02	589.3	0.05	N/A	34.59	0
23	2022-02-09	589.3	0.16	N/A	1.74	0.0003
23	2022-02-14	589.3	0.01	0.04	7.03	0
25	2022-02-15	248	0.01	0.03	3.89	0
26	2022-02-15	589.3	0.22	0.32	12.15	0.0004
26	2022-02-17	56.9	0.19	N/A	25.65	0.003
26	2022-02-21	108	0.35	0.36	38.78	0.0035
26	2022-02-22	19.9	0.05	0.18	16.21	0.0031
26	2022-02-25	77.2	0.05	N/A	48.85	0.0002
26	2022-02-25	492.3	0.07	N/A	1.56	0.0002
M1	2022-09-21	185.33	0.14	0.34	7.08	0.0009
M1	2022-09-22	28.07	0.04	N/A	13.68	0.0011
M1	2022-09-23	48.9	0.07	0.07	10.54	0.0012
M1	2022-09-26	93.03	0.13	0.2	7.98	0.0015
M1	2022-09-27	132.87	0.12	N/A	14.34	0.0009
M3	2022-09-19	23.37	0.04	N/A	6.34	0.0018
M3	2022-09-21	118.3	0.06	0.76	6.07	0.001
M3	2022-09-22	110.07	0.17	2.15	12.65	0.0019
M3	2022-09-22	124.8	0.29	2.15	7.98	0.0022
M3	2022-09-23	187.93	0.38	0.49	10.77	0.0023
M4	2022-09-19	14.13	0.03	N/A	4.32	0.0013
M4	2022-09-22	101.7	0.11	N/A	15.49	0.0012
M4	2022-09-23	37.6	0.04	N/A	29.67	0.001
M5	2022-09-21	196.43	0.05	0.21	6.71	0.0003
M5	2022-09-23	167.5	0.05	0.3	10.27	0.0003
M5	2022-09-26	191.9	0.05	0.2	7.78	0.0002
M5	2022-09-26	108.27	0.04	0.2	10.7	0.0004
M5	2022-09-27	176.7	0.09	0.45	12.49	0.0005
M6	2022-09-22	47.1	0.09	N/A	32.44	0.0017
M6	2022-09-22	109.63	0.15	N/A	22.57	0.0013
M6	2022-09-23	89.5	0.19	0.42	9.87	0.002
M6	2022-09-26	186.83	0.21	0.38	6.27	0.0011
M6	2022-09-27	40.17	0.07	0.26	34.59	0.0016
M8	2022-09-20	158.3	0.29	0.95	5.4	0.0024
M8	2022-09-23	59.07	0.12	N/A	9.7	0.0023
M9	2022-09-20	66.73	0.05	0.17	21.79	0.0003

Table S1 The table summarizes the subset of collected videos that are used in the analysis. Measurements have been rounded for space concerns. Mass differences are per day, while all other measurements are per recording.
