# Supplementary material for: Feeding rate in adult Manduca sexta is unaffected by proboscis submersion depth
Source: PLoS One. 2024 May 29;19(5):e0302536. doi: 10.1371/journal.pone.0302536 (PMC11135714; doi:10.1371/journal.pone.0302536)
Supplement: S2 Table — The table summarizes the subset of collected videos that were excluded from the analysis and gives the reasoning for why they were excluded. Measurements have been rounded for space reasons. The entries that resemble duplicates originate from distinct recordings on the same day from different moths. Mass differences are per day, while other measurements are per recording. (PDF) [file pone.0302536.s002.pdf]

moth ID	date	Reason for exclusion	Recording Duration (s)	intake volume for recording (mL)	mass change for moth and date (g)
23	2022-02-15	almost no nectar ingestion occurs, feeding behavior likely not present	508.6	0.01	0.02
22	2022-02-07	excessive visibility of experimenter (100% of frames)	55.1	0.02	N/A
N/A	2022-01-28	subject deceased, feeding behavior unlikely	20	N/A	N/A
M8	2022-09-20	flower is not vertical and flower is not in stationary position	147.7	0	0.95
26	2022-02-16	flower design differs from protocol	146.7	N/A	0.38
22	2022-02-11	flower is not always in focus, excessive visibility of experimenter (>10% of all frames)	589.2	0.04	N/A
22	2022_02_09	flower is not stationary, excessive visibility of experimenter (>10% of all frames)	576.1	0	N/A
23	2022-02-11	minimal nectar ingestion,unusual proboscis position, inducement of feeding behavior attempted likely to produce misleading data	181.8	0.04	N/A
22	2022-01-28	minimal nectar ingestion, unusual proboscis position, feeding behavior likely not present	4	0.01	N/A
26	2022-02-23	minimal nectar ingestion, unusual proboscis possition, inducement of feeding behavior likely to produce misleading data, excessive visibility of experimenter (>10% of all frames)	123.9	0.04	0.11
22	2022-02-04	flower is not stationary	180	0.12	N/A
22	2022-02-04	nectar content in resevoir in excess of protocol, nectar ingestion not measurable	46.8	0.58	N/A
22	2022-01-28	produces linear algebra error during analysis	5	0	N/A

Table S6 Unsuitable recordings are excluded from analysis. The table summarizes the subset of collected videos that were excluded from the analysis and gives the reasoning for why they were excluded. Measurements have been rounded for space reasons. The entries that resemble duplicates originate from distinct recordings on the same day from different moths. Mass differences are per day, while other measurements are per recording.
