# Supplementary figures and images for: Feeding rate in adult Manduca sexta is unaffected by proboscis submersion depth
Source: PLoS One. 2024 May 29;19(5):e0302536. doi: 10.1371/journal.pone.0302536 (PMC11135714; doi:10.1371/journal.pone.0302536)

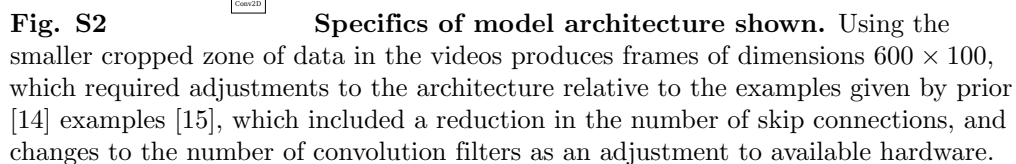

Supplement: S1 Fig — Using the smaller cropped zone of data in the videos produces frames of dimensions 600 × 100, which required adjustments to the architecture relative to the examples given by prior [14] examples [15], which included a reduction in the number of skip connections, and changes to the number of convolution filters as an adjustment to available hardware. (PDF) [file pone.0302536.s003.pdf]
