# Supplementary material for: Feeding rate in adult Manduca sexta is unaffected by proboscis submersion depth
Source: PLoS One. 2024 May 29;19(5):e0302536. doi: 10.1371/journal.pone.0302536 (PMC11135714; doi:10.1371/journal.pone.0302536)
Supplement: S2 Fig — Bottom row shows strip plots of all available data, top row shows line plots aggregating the same by moth ID. Left and right columns have been split to elide a period of several months where no data were collected. The date, and therefore the flower design, does not appear to have a consistent effect on the drinking rate of the moths. If the refinements had changed the behavior, we would expect to see general upward trends in the median drinking rate as the date progresses. (PDF) [file pone.0302536.s004.pdf]

Fig. S3

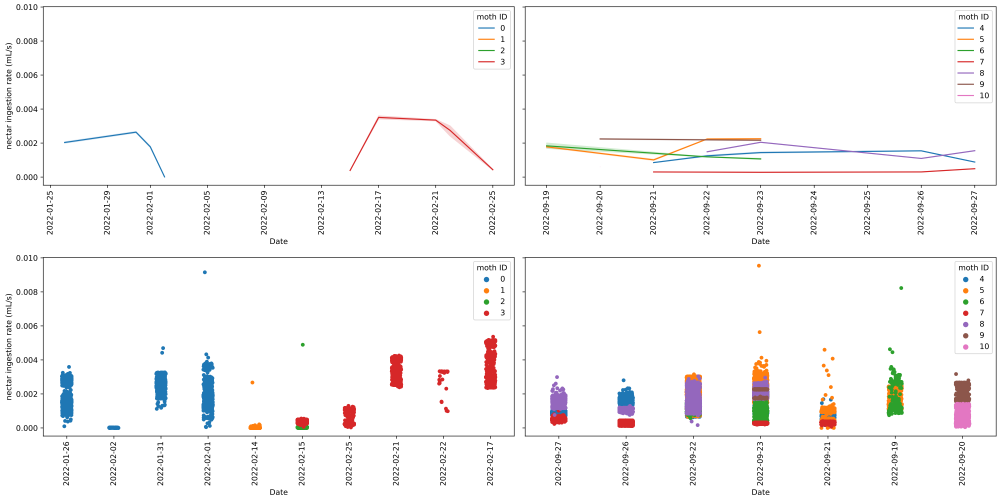

Drinking rates are not influenced by date measured, and therefore independent of artificial flower design. Bottom row shows strip plots of all available data, top row shows line plots aggregating the same by moth ID. Left and right columns have been split to elide a period of several months where no data were collected. The date, and therefore the flower design, does not appear to have a consistent effect on the drinking rate of the moths. If the refinements had changed the behavior, we would expect to see general upward trends in the median drinking rate as the date progresses.
