# Supplementary material for: Feeding rate in adult Manduca sexta is unaffected by proboscis submersion depth
Source: PLoS One. 2024 May 29;19(5):e0302536. doi: 10.1371/journal.pone.0302536 (PMC11135714; doi:10.1371/journal.pone.0302536)
Supplement: S3 Fig — Points are colored by the moth ID for the recording and icons denote whether the moth flies in the recording or not. A linear model is drawn on the graph with a 95% confidence interval. Recordings with curled proboscises visible have been excluded. The model is dependent on the outlier at the bottom left, and excluding it causes the model to be statistically insignificant (p = 0.09 >0.05), for which the null hypothesis must be presumed. (PDF) [file pone.0302536.s005.pdf]

Fig. S4

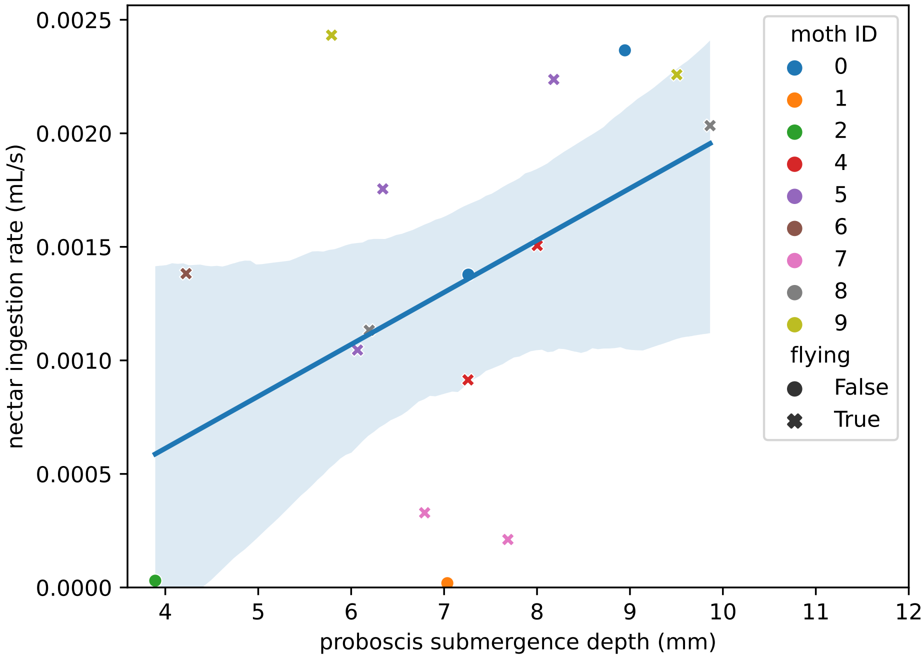

Submergence versus drinking rate remains independent when submergence depth is restricted to not exceed 10 mm. Points are colored by the moth ID for the recording and icons denote whether the moth flies in the recording or not. A linear model is drawn on the graph with a 95% confidence interval. Recordings with curled proboscises visible have been excluded. The model is dependent on the outlier at the bottom left, and excluding it causes the model to be statistically insignificant ($p = 0.09 > 0.05$), for which the null hypothesis must be presumed.
