# Supplementary material for: Feeding rate in adult Manduca sexta is unaffected by proboscis submersion depth
Source: PLoS One. 2024 May 29;19(5):e0302536. doi: 10.1371/journal.pone.0302536 (PMC11135714; doi:10.1371/journal.pone.0302536)
Supplement: S4 Fig — Residuals of nectar ingestion rate are graphed against proboscis submergence depth. (PDF) [file pone.0302536.s006.pdf]

Fig. S5

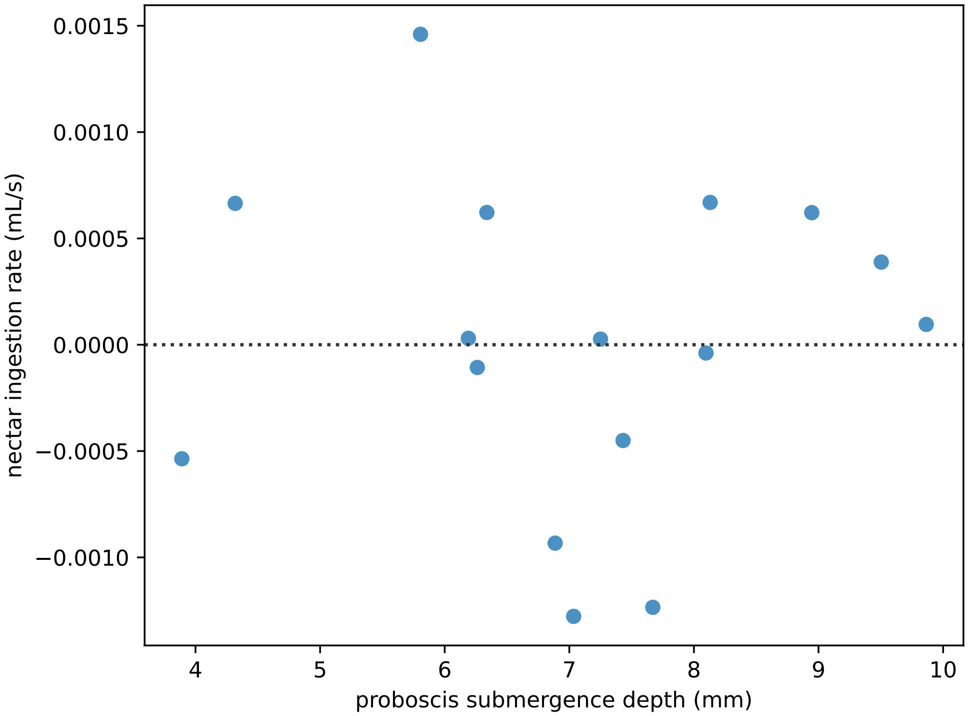

No pattern in the residuals of drinking rate against submergence not exceeding 10 mm. Residuals of nectar ingestion rate are graphed against proboscis submergence depth.
