# Supplementary material for: Feeding rate in adult Manduca sexta is unaffected by proboscis submersion depth
Source: PLoS One. 2024 May 29;19(5):e0302536. doi: 10.1371/journal.pone.0302536 (PMC11135714; doi:10.1371/journal.pone.0302536)
Supplement: S5 Fig — A scatterplot showing all measured proboscis submergences against all measured nectar ingestion rates by moth. As long as contact is made with the fluid, drinking can occur. The depth of submergence does not predict the rate of nectar ingestion, nor vice versa. Multiple nectar intake rates are observed at a given submergence depth, and multiple submergence depths are observed for almost any given intake rate. (PDF) [file pone.0302536.s007.pdf]

Fig. S7

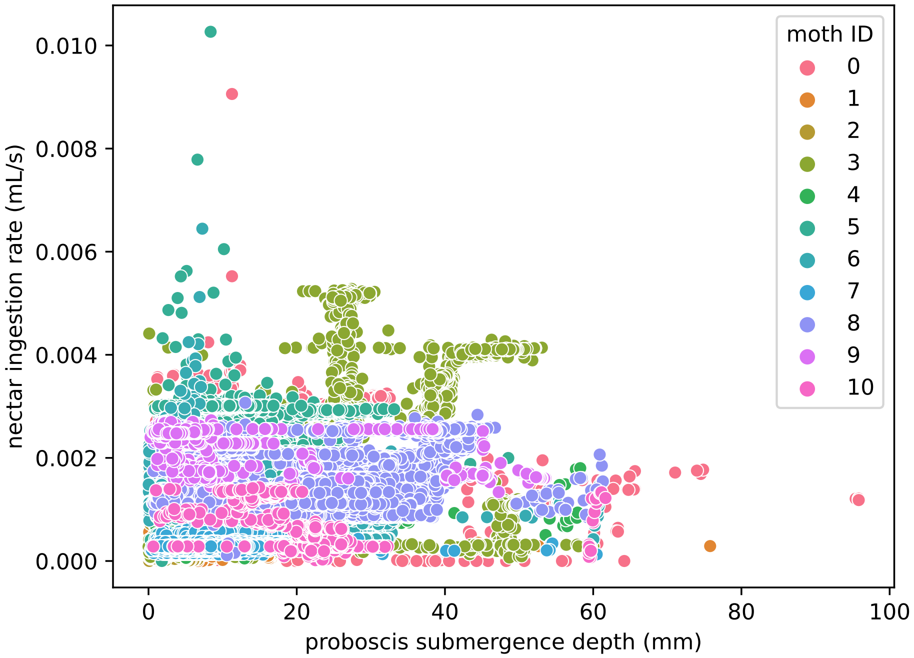

No relationship between nectar ingestion rate and proboscis submergence depth exists in raw data. A scatterplot showing all measured proboscis submergences against all measured nectar ingestion rates by moth. As long as contact is made with the fluid, drinking can occur. The depth of submergence does not predict the rate of nectar ingestion, nor vice versa. Multiple nectar intake rates are observed at a given submergence depth, and multiple submergence depths are observed for almost any given intake rate.
